# Protheseninfektionen und die zunehmende Bedeutung psychologischer Komorbiditäten: Eine epidemiologische Analyse für Deutschland von 2009 bis 2019

**DOI:** 10.1007/s00132-021-04088-7

**Published:** 2021-03-09

**Authors:** Nike Walter, Markus Rupp, Thilo Hinterberger, Volker Alt

**Affiliations:** 1 Klinik und Poliklinik für Unfallchirurgie, Universitätsklinikum Regensburg, Franz-Josef-Strauß-Allee 11, 93053 Regensburg, Deutschland; 2 Abteilung für Psychosomatische Medizin, Universitätsklinikum Regensburg, Franz-Josef-Strauß-Allee 11, Regensburg, 93053 Deutschland

**Keywords:** Periprothetische Gelenkinfektion, Psychologische Symptome, Epidemiologie, Nebenerkrankungen, Gelenkersatz, Periprosthetic joint infection, Psychological symptoms, Epidemiology, Comorbidities, Total joint arthroplasty

## Abstract

**Hintergrund:**

Die periprothetische Gelenkinfektion (PJI) ist eine gefürchtete Komplikation in der Orthopädie und Unfallchirurgie. Ein potenzieller Anstieg an PJI-Diagnosen, insbesondere in Verbindung mit psychologischen Komorbiditäten, kann zu einer besonderen Herausforderung für Akteure im Gesundheitswesen werden. Bisher ist die Prävalenz für Deutschland unbekannt. Dies erschwert es, den zukünftigen Behandlungsbedarf abzuschätzen und Entwicklungen vorherzusehen, die durch eine Anpassung von Präventions- und Therapiemaßnahmen beeinflusst werden können.

**Ziel der Arbeit:**

Die vorliegende Arbeit gibt eine detaillierte Übersicht über die Epidemiologie periprothetischer Gelenkinfektionen und psychologischer Komorbiditäten.

**Material und Methoden:**

Ein Datensatz vom Statistischen Bundesamt (Destatis) aus jährlichen, deutschlandweiten ICD-10-Diagnosekodes von 2009 bis 2019 wurde analysiert. Prävalenzraten des Kodes „T84.5 – Infektion und entzündliche Reaktion durch eine Gelenkendoprothese“ wurden nach Altersgruppe, Geschlecht und in Verbindung mit einer Nebendiagnose des Kapitels F quantifiziert und aufgeschlüsselt.

**Ergebnisse:**

Seit 2009 steigen die PJI-Diagnosen kontinuierlich an, die Häufigkeit war im Jahr 2018 rückläufig. Im Jahr 2019 wurden 16.174 Fälle entsprechend einer Prävalenz von 23,8/100.000 Einwohner verzeichnet. Eine Entwicklung hinsichtlich mehr Diagnosen bei älteren Patienten wurde evident. Ein Viertel aller Patienten wiesen eine Nebendiagnosen im Bereich psychischer Störungen und Verhaltensstörungen auf, wobei sich die Anzahl an Patienten mit psychologischen Komorbiditäten im letzten Jahrzehnt verdoppelte.

**Schlussfolgerung:**

Richtlinien zu Präventionsstrategien und psychologische Unterstützungsangebote sollten in der Unfallchirurgie implementiert werden.

## Abstract (en)

**Background:**

Periprosthetic joint infection (PJI) is a devastating complication in orthopedics and trauma surgery. A potential increase in PJI diagnoses, especially in combination with psychological comorbidities may become a significant challenge for healthcare stakeholders. So far, the prevalence for Germany is unknown, which makes it difficult to estimate future treatment demand or to predict developments that can be influenced by adapting prevention and treatment measures.

**Objectives:**

The present study provides a detailed overview of the epidemiology of periprosthetic joint infections and psychological comorbidities.

**Materials and methods:**

A dataset provided by the Federal Statistical Office (Destatis) consisting of annual, Germany-wide ICD-10 diagnosis codes from 2009 through 2019 was analyzed. Incidences of the code “T84.5—Infection and inflammatory reaction due to joint arthroplasty” were quantified, and a detailed breakdown of this data by age group and gender was performed. Prevalence rates of secondary diagnoses of the ICD-10 chapter F were determined.

**Results:**

PJI diagnoses have been steadily increasing since 2009. The incidence declined in 2018. In 2019, 16,174 cases were registered, corresponding to a prevalence of 23.8/100,000 population. A trend towards more diagnoses in elderly patients became evident. Twenty-five percent of all patients had a secondary diagnosis regarding mental and behavioural disorders. The number of patients with psychological comorbidities doubled in the last decade.

**Conclusions:**

Guidelines on prevention strategies as well as psychological support services should be implemented in trauma surgery.

Periprothetische Gelenkinfektionen (PJI) sind eine gefürchtete Komplikation in der Orthopädie und Unfallchirurgie. Ein potenzieller Anstieg an PJI-Diagnosen, insbesondere in Verbindung mit psychologischen Komorbiditäten, kann zu einer besonderen Herausforderung für Akteure im Gesundheitswesen werden. Bisher ist die Prävalenz für Deutschland unbekannt. Dies erschwert es, den zukünftigen Behandlungsbedarf abzuschätzen und Entwicklungen vorherzusehen, die durch eine Anpassung von Präventions- und Therapiemaßnahmen beeinflusst werden können. In diesem Beitrag wird eine epidemiologische Analyse berichtet.

## Hintergrund und Fragestellung

Der endoprothetische Gelenkersatz ist ein lebensverbesserndes Verfahren für Millionen von Menschen auf der ganzen Welt, der neben einer Schmerzlinderung auch die Funktion des betroffenen Gelenkes wiederherstellen kann. In Deutschland gehört das Implantieren einer Knie- oder Hüftendoprothese zu den häufigsten Eingriffen, wobei ein Anstieg der Operationszahlen um bis zu 45 % für das Jahr 2040 prognostiziert wurde [24]. Eine gefürchtete Komplikation in der Orthopädie und Unfallchirurgie ist die periprothetische Gelenkinfektion (PJI). Für Patienten stellt eine Protheseninfektion eine hohe Belastung dar und schwerwiegende Einschränkungen sind oft – trotz modernster und interdisziplinärer Behandlungskonzepte – nicht zu vermeiden. Folgen sind lange Krankenhausaufenthalte mit mehrfachen Operationen, einschließlich Entfernung des Implantats und Resektion des infizierten Knochens, Immobilität, erhöhte Gabe von lokalen und systemischen Antibiotika, häufig verbunden mit Nebenwirkungen und weitere sozioökonomischen Folgen. Darüber hinaus ist eine PJI potenziell lebensbedrohlich mit einer 3,2–3,7fach erhöhten Sterblichkeitsrate im Vergleich zu Patienten, bei denen im Verlauf eines Gelenkersatzes keine Infektion auftritt [26]. Die Behandlung ist komplex und abhängig von diversen Faktoren, wie Dauer der Infektion, zugrundeliegende Erreger sowie Zustand der implantierten Prothese und der Weichteile. Mögliche Optionen sind das chirurgische Debridement mit Implantaterhalt und Antibiotikatherapie, eine einzeitige Revision oder ein zweizeitiger Prothesenwechsel, bei dem eine neue Revisionsendoprothese nach einem implantatfreien Intervall eingesetzt wird. Bei einer beträchtlichen Anzahl von Patienten wird die Reimplantation nicht als durchführbar erachtet, sodass nur eine Gelenkversteifung durch knöcherne Fusion oder ein Arthrodeseimplantant, die Schaffung einer Girdlestone-Situation, sowie die Amputation der affektierten Gliedmaßen als Therapiealternativen verbleiben.

Durch die schlechteren Behandlungsergebnisse im Vergleich zu Fällen, die keine Revision benötigen, haben PJI, verbunden mit Schmerzen, Bewegungseinschränkungen sowie Verlust der Mobilität und Selbstständigkeit, tiefgreifende Auswirkungen auf die Lebensqualität betroffener Patienten und führen zu einem hohen Maß an psychologischem Stress [7, 9, 16, 17, 23, 32].

Durch die Zunahme der Primärimplantationen und PJI-spezifischen Risikofaktoren wie Übergewicht oder Diabetes mellitus kann ebenfalls ein erhöhtes Auftreten von Gelenksinfektionen erwartet werden [8, 15, 30]. Während ansteigende PJI-Inzidenzen für die USA, Dänemark, Finnland, Norwegen, Schweden, Neuseeland, England und Irland beschrieben wurden [4, 12, 27], ist die Prävalenz für Deutschland unbekannt. Dieses erschwert es, den zukünftigen Bedarf abzuschätzen und Entwicklungen vorherzusehen, die durch eine Anpassung der Präventions- und Therapiemaßnahmen beeinflusst werden könnten. Neben weiteren somatischen Nebenerkrankungen, wie rheumatoide Arthritis und Hyperthyreose [10, 29], die das Auftreten einer Infektion beeinflussen, spielen auch psychologische Komorbiditäten eine entscheidende Rolle. So erhöht beispielsweise Tabakkonsum, bei Abhängigkeitssyndrom und schädlichem Gebrauch kodiert unter Kapitel F17 des ICD-10, das Infektionsrisiko nach Gelenkersatz signifikant [5]. Auch die Nebendiagnose Depression führt zu erhöhtem Infektrisiko nach Gelenkersatz und ist assoziiert mit schlechteren klinischen Ergebnissen [2, 3, 22], wobei jedoch bisher das Potenzial von psychologisch unterstützenden Interventionen nicht untersucht wurde [11].

Daher ist das Ziel der vorliegenden Arbeit die Beantwortung der folgenden Forschungsfragen:

- Wie haben sich die PJI-Inzidenzen im Laufe des letzten Jahrzehnts entwickelt?
- Welchen Einfluss haben die Faktoren Alter und Geschlecht auf die Häufigkeit der Diagnose?
- Wie hat sich der Anteil an Patienten mit psychologischen Nebenerkranken in den letzten 10 Jahren entwickelt?
- Welche psychologischen Komorbiditäten kommen am häufigsten bei PJI vor?

## Studiendesign und Untersuchungsmethoden

Ein Datenset, bestehend aus jährlichen ICD-10-Diagnosekodes aus deutschen medizinischen Einrichtungen von 2009 bis 2019, wurden vom Statistischen Bundesamt (Destatis) zur Verfügung gestellt. Zunächst wurde die Anzahl der ICD-10-Diagnosen mit dem Kode „T84.5 – Infektion und entzündliche Reaktion durch eine Gelenkendoprothese“ nach Altersgruppe in 10-Jahres-Inkrementen und Geschlecht quantifiziert. Die Prävalenzraten wurden auf Basis der von Destatis bereitgestellten historischen Bevölkerung Deutschlands älter als 20 Jahre berechnet [28]. Dabei wurde für jedes Jahr des Zeitraums 2009 bis 2019 die Anzahl der Einwohner in jedem der 16 Bundesländer nach Geburtsjahrgängen betrachtet. Stichtag eines jeden Jahres war der 31. Dezember. Zusätzlich wurde die Anzahl der stationären Patienten mit der Hauptdiagnose „T84.5“ und einer Nebendiagnose des Kapitels F des ICD-10, Psychische und Verhaltensstörungen, bestimmt. Die prozentuale Verteilung der Nebendiagnosen wurden unterteilt für die Diagnosen „F0, F1, F2, F3, F4, F5, F6, F7, F8“ und „F9“ (Tab. 1). Darüberhinausgehende, weitere Nebendiagnosen wurden in der Auswertung nicht berücksichtigt.

**Table 1:** 

ICD-10-Kode	Beschreibung	Beispielerkrankungen
T84.5	Infektion und entzündliche Reaktion durch eine Gelenkendoprothese	Periimplantäre (implantatassoziierte) Infektion
F0	Organische, einschließlich symptomatischer psychischer Störungen	Demenz bei Alzheimer-Krankheit, vaskuläre Demenz, Delir, nicht durch Alkohol oder andere psychotrope Substanzen bedingt
F1	Psychische und Verhaltensstörungen durch psychotrope Substanzen	Psychische und Verhaltensstörungen durch Alkohol, Cannabinoide, Tabak, Sedativa oder Hypnotika
F2	Schizophrenie, schizotype und wahnhafte Störungen	Schizophrenie, schizotype Störung, anhaltende wahnhafte Störung
F3	Affektive Störungen	Manische Episode, bipolare affektive Störung, depressive Episode
F4	Neurotische, Belastungs- und somatoforme Störungen	Phobische Störungen, Zwangsstörungen, dissoziative Störungen
F5	Verhaltensauffälligkeiten mit körperlichen Störungen und Faktoren	Essstörungen, nichtorganische Schlafstörungen
F6	Persönlichkeits- und Verhaltensstörungen	Spezifische Persönlichkeitsstörungen: paranoide, dissoziale, emotional instabile (impulsiver Typ, Borderline-Typ)
F7	Intelligenzstörungen	Leichte Intelligenzminderung, schwere Intelligenzminderung
F8	Entwicklungsstörungen	Umschriebene Entwicklungsstörungen des Sprechens und der Sprache, umschriebene Entwicklungsstörungen motorischer Funktionen
F9	Verhaltens- und emotionale Störungen mit Beginn in der Kindheit und Jugend	Hyperkinetische Störungen, Ticstörungen

## Ergebnisse

**Figure 1:**
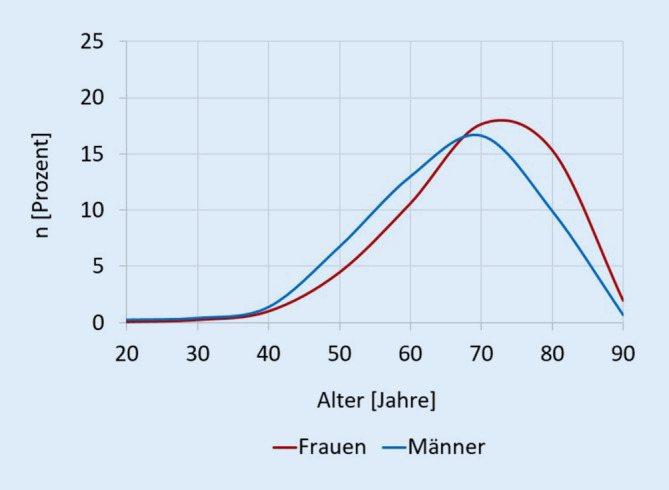

Seit dem Jahr 2009 konnte ein kontinuierlicher Anstieg der PJI-Diagnosen verzeichnet werden, die mit 18.797 Fällen im Jahr 2017 ein Maximum erreichten, woraufhin die Häufigkeit abfiel auf 16.174 Fälle im Jahr 2019 mit einer Inzidenz von 23,8/100.000 Einwohner. Im Vergleich zum Jahr 2009 betrug die relative Veränderung +3,54 % (Tab. 2). Die Geschlechterverteilung blieb über das letzte Jahrzehnt nahezu konstant. Das Frauen-Männer-Verhältnis betrug 52,1/47,9 % im Jahr 2009 bzw. 51,3/48,7 % im Jahr 2019. Im Jahr 2019 waren Frauen im Alter von 70–79 Jahren am häufigsten betroffen (17,64 %), gefolgt von der Altersgruppe 80–89 Jahre (15,35 %) und 60–69 Jahre (10,59 %). Der größte Anteil der PJI-Diagnosen bei Männern im Jahr 2019 wurde für die Altersgruppe 70–79 Jahre berechnet (16,61 %), gefolgt von Patienten im Alter 60–69 Jahren (12,96 %) und 80–89 Jahren (9,9 %) (Abb. 1). Insgesamt konnte eine Entwicklung hinsichtlich mehr PJI-Diagnosen bei älteren Patienten beobachtet werden, mit einem Anstieg von 25,70 % der Fälle in der Altersgruppe 80–89 Jahre bei Frauen und +71,24 % Veränderung bei Männern im Alter 80–89 Jahre zwischen 2009 und 2019. Die Fallzahlen für das Altersinkrement älter als 90 Jahre stiegen für Frauen ums 2,3fache, bei Männern gab es eine 2,8fache Zunahme.

**Table 2:** 

Jahr	PJI-Diagnosen	Relative Veränderung zu 2009	Deutsche Population älter als 20 Jahre	PJI-Inzidenz/100.000 Einwohner	Nebendiagnosen Kapitel F	Nebendiagnosen prozentual
2009	15.621	–	66.400.066	23,5	2846	18,22
2010	16.676	+6,75	66.549.975	25,1	3337	20,01
2011	17.267	+10,54	65.398.514	26,4	3517	20,37
2012	17.537	+12,27	65.665.069	26,7	3595	20,50
2013	18.124	+16,02	65.943.867	27,5	3924	21,65
2014	18.417	+17,90	66.677.665	27,6	4005	21,75
2015	18.613	+19,15	67.097.676	27,7	4198	22,55
2016	18.543	+18,71	67.440.230	27,5	4418	23,83
2017	18.797	+20,33	67.540.025	27,8	4495	23,91
2018	18.283	+17,04	67.724.921	27,0	4316	23,61
2019	16.174	+3,54	67.864.036	23,8	4043	25,00

**Figure 2:**
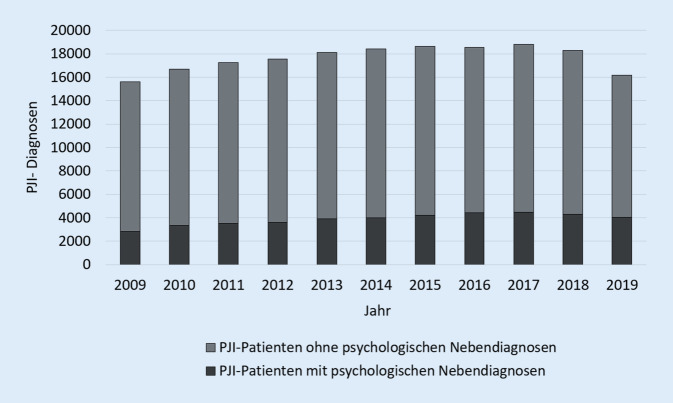

**Figure 3:**
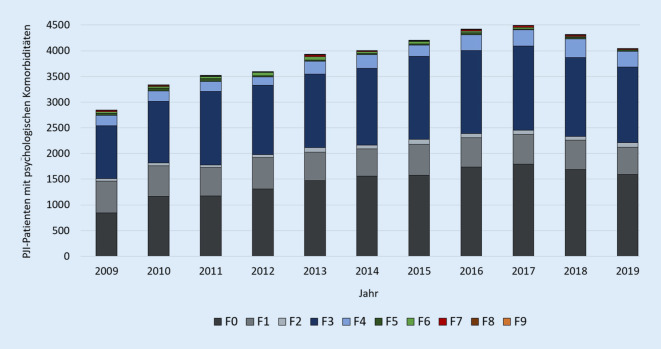

Im Jahr 2019 wurden 25 % der PJI-Patienten mit einer psychischen Nebenerkrankung registriert. Die Häufigkeit der Kapitel-F-Nebendiagnosen stieg seit 2009 stetig an, mit einer Zunahme von 42,1 % von 2846 Fälle auf 4043 Fälle im Jahr 2019 (Tab. 2; Abb. 2). Insbesondere die Nebendiagnose F0, organische, einschließlich symptomatischer psychischer Störungen, erhöhte sich in den letzten 10 Jahren um 88,05 % und betraf mit 1589 Fällen im Jahr 2019 39,3 % aller PJI-Patienten mit psychologischen Komorbiditäten. Ein Anstieg wurde ebenfalls bezüglich des ICD-Kodes F3, affektive Störungen, verzeichnet (+43,29 %). Mit 1473 Fällen im Jahre 2019 waren 36,43 % der Patienten mit Nebendiagnosen betroffen. Am dritthäufigsten kamen psychische und Verhaltensstörungen durch psychotrope Substanzen (F1) mit 13,13 % und 513 Fällen im Jahr 2019 bei PJI-Patienten vor (Tab. 3; Abb. 3).

**Table 3:** 

Diagnose	Prozentuale Häufigkeit	Relative Veränderung zu 2009
F0	39,30	+88,05
F1	13,13	−14,49
F2	2,25	+85,71
F3	36,43	+43,29
F4	7,62	+57,95
F5	0,40	−65,22
F6	0,40	−48,39
F7	0,40	−20,00
F8	0,0	0,0
F9	0,07	−72,73

## Diskussion

In dieser Studie wurde die Häufigkeit von periprothetischen Gelenkinfektionen in Verbindung mit psychologischen Komorbiditäten bestimmt. Die deutlich geringere Anzahl der Fälle im Jahr 2019 im Vergleich zu den Vorjahren, lässt darauf schließen, dass vereinheitlichte Richtlinien zu Präventionsstrategien wirksam werden [21, 25]. Ein Großteil der Fälle war in der Altersgruppe 70–79 Jahre verzeichnet, wobei eine Entwicklung hinsichtlich mehr PJI-Diagnosen bei älteren Patienten evident wurde. Frauen und Männer waren nahezu gleich verteilt betroffen. Ein Viertel aller Patienten wiesen eine Nebendiagnosen im Bereich psychischer Störungen und Verhaltensstörungen, Kapitel F des ICD-10, auf, wobei organische, einschließlich symptomatischer psychischer Störungen und affektive Störungen am häufigsten auftraten. Die Anzahl an Patienten mit psychologischen Komorbiditäten verdoppelte sich im letzten Jahrzehnt. Da die Einführung des G‑DRG-Systems bereits im Jahr 2004 umgesetzt wurde, kann der Einfluss im Sinne erhöhter Fallzahlkodierungen als gering erachtet werden. Anhand des analysierten Datensatzes kann keine Aussage über die Behandlungsstrategie getroffen werden. Unklar ist daher, ob PJI-Patienten mit Nebendiagnosen psychologische Betreuung erhielten. Nichtsdestotrotz spricht der Anstieg für eine Implementierung von psychologischen Screenings und den Einsatz von Fragebögen, wie etwa einem zeitsparenden ICD-10-basierten Symptomrating [31] sowie psychischer Unterstützungsangebote in der Orthopädie und Unfallchirurgie. Obwohl der Bedarf an psychologischer Unterstützung explizit von Patientenseite benannt wurde [13, 17], ist das mentale Wohlbefinden der Patienten bisher kaum in den Fokus der Chirurgie gerückt. Wissenschaftlich ist die Erkenntnis, dass die psychische Verfassung einen signifikanten Einfluss auf das Behandlungsergebnis hat, gut fundiert [2, 6, 20, 22]. Nichtsdestotrotz wurde in einer systematischen Literaturarbeit, in der 4213 Artikel zur Behandlung von PJI identifiziert wurden, keine Studie gefunden, die Auswirkungen psychologischer Interventionen untersuchten [11]. Wenn eine bessere Versorgung unserer Patienten erreicht werden soll, ist interdisziplinäre Zusammenarbeit geboten und Psychologen oder Psychiater sollten nicht im Behandlungsteam fehlen [14]. Gerade unter den aktuellen Umständen der COVID-19-Pandemie ist mit einer weiteren Zunahme psychischer Erkrankungen zu rechnen. So berichteten Ohliger und Kollegen von einer höheren Prävalenz psychiatrischer Erkrankungen bei orthopädischen Patienten während der COVID-19-Pandemie (76 von 298 Patienten [26 %] vs. 110 von 255 Patienten [43 %], *p* < 0,001) im Vergleich zu 2019 in den USA [19]. Darüber hinaus betonten mehrere Studien ein geringeres psychisches Wohlbefinden in der Allgemeinbevölkerung im Vergleich zur Zeit vor COVID-19 sowie eine erhöhte Symptombelastung bei Patienten mit vorbestehenden psychiatrischen Erkrankungen [1, 33]. Da mit langanhaltenden Belastungen gerechnet werden muss, ist es dringend erforderlich, die Förderung der psychischen Gesundheit und die Prävention nicht nur in der Orthopädie und Unfallchirurgie, sondern auch global zu verstärken [18]. Konkrete Möglichkeiten zur Verbesserung der Lebensqualität beinhalten die Implementierung von psychologischen Beratungsstellen, Rauchentwöhnungsprogrammen und Ernährungsberatung. Ebenfalls sollten mehr Ressourcen für Sozialdienste in Kliniken zur Verfügung gestellt werden, damit Patienten persönlich betreut werden können und auch Angehörige adäquate Orientierungshilfe bekommen. Angebote wie Selbsthilfegruppe, Gesprächskreise und Kurse zu Entspannungsverfahren sollten verstärkt bereitgestellt und Patienten umfassend über diese informiert werden.

## Fazit für die Praxis

- Ein Screening nach psychologischen Symptomen sollte in der Orthopädie und Unfallchirurgie implementiert werden.
- Die interdisziplinäre Zusammenarbeit mit Psychiatern wird empfohlen.
- Psychologische Unterstützungsangebote zur Verbesserung der Lebensqualität von Patienten mit periprothetischen Gelenkinfektionen werden benötigt

## Abkürzungen

COVID-19: „Coronavirus disease 2019“
G‑DRG: German Diagnosis Related Groups
ICD: International Statistical Classification of Diseases and Related Health Problems
PJI: Periprothetische Gelenkinfektion
